# The American Journal of Dental Science

**Published:** 1867-06

**Authors:** 


### THE AMERICAN JOURNAL OF DENTAL SCIENCE.

There are several journals in America devoted to Dental science; but this is *the* "American Journal." How familiar the name sounds! As I write, I fancy myself fanned by the great angel-wings of the "lamented"—why no!—of the *sainted* Harris.

The "American Journal" is revived—a monthly, at $3 per annum, and is edited by Professors Piggot and Gorgas. The first number makes a fine appearance, and has a good variety of able and well written communications and editorials. Professor Piggot has a large editorial experience, and a fine literary reputation. Professor Gorgas is a clear and forcible writer. We never felt content with the suspension of the "American Journal." Its apparent demise suggested, all too painfully, that the "Register" might share the same fate, if somebody should die. We feel much better now. The "Register" had got very tired being the oldest Dental journal.

Reader, subscribe for the *Journal.* Your money will be well invested. W.

